# Pediatric Oronasopharyngeal Stricture– A Rare Surgical Complication of Adeno-Tonsillectomy Abstract

**DOI:** 10.1007/s12070-023-03694-5

**Published:** 2023-03-20

**Authors:** Vijendra S Shenoy, Rakshitha Samanth, Navya Parvathareddy, KV APOORVA

**Affiliations:** Dept of ENT and Head & neck surgery, Kasturba Medical College, Mangalore, Manipal Academy of Higher Education, Mangalore, India

**Keywords:** Stricture, Airway, Coblator, Adenotonsillectomy, Palatoplasty

## Abstract

Introduction: Oronasopharyngeal stricture is a rare sequel of oropharyngeal surgical procedure which can cause swallowing difficulty, dyspnea, sleep related breathing disorders, incompetence at the velopharynx due to soft palatal adherence. Case: Here we present a case of nine-year-old girl who presented to us with complains of nasal obstruction since 3 months and symptoms of sleep disordered breathing. She underwent adenotonsillectomy 5 years ago and coblator assisted palatoplasty and stricture release 4 months ago. On examination adenoid facies was noted, postpalatoplasty and tonsillectomy status with stricture between the anterior and posterior tonsillar pillars. Soft palate was stuck to posterior pharyngeal wall. Computed tomography scan was done which depicted homogenously enhancing soft tissue density causing complete nasopharyngeal airway obstruction. Oronasopharyngeal stricture release was performed under general anesthesia. Infant feeding tube was inserted through the nasal cavity and introduced through the oropharynx into exterior and was tied externally which was removed on postoperative day 5. Child is on regular follow up since 5 months and asymptomatic. Discussion: Oropharyngeal stricture is reported as a rare sequela of adenotonsillectomy. Many surgical techniques have been reported for release of similar oropharyngeal and nasopharyngeal stricture patterns such as triamcinolone injection, manual dilation method, plasma hook method, division and skin grafting, local flaps like pharyngeal or palatal mucosal flaps, and free flap techniques. Conclusion: Utmost care must be taken during the surgical procedure of oropharynx to prevent collateral damage to mucosal surfaces which can result in such devastating complications.

**Supplementary Information:**

The online version contains supplementary material available at 10.1007/s12070-023-03694-5.

## Introduction

Oronasopharyngeal stricture is a rare sequel of oropharyngeal surgical procedure which can cause swallowing difficulty, dyspnea, sleep related breathing disorders, incompetence at the velopharynx due to soft palatal adherence [1]. According to literature, this case is the first case report who had oronasopharyngeal stricture after routine adenotonsillectomy with nil other risk factors and recurred after coblator assisted stricture release.

The 1st repair of nasopharyngeal stricture was done in the year 1896 by Nichols [2]. Many surgical techniques have been reported for release of similar oropharyngeal and nasopharyngeal stricture patterns such as triamcinolone injection, manual dilation method, division and skin grafting, local flaps like pharyngeal or palatal mucosal flaps, and free flap techniques [3].

## Case Report

**Fig. 1:**
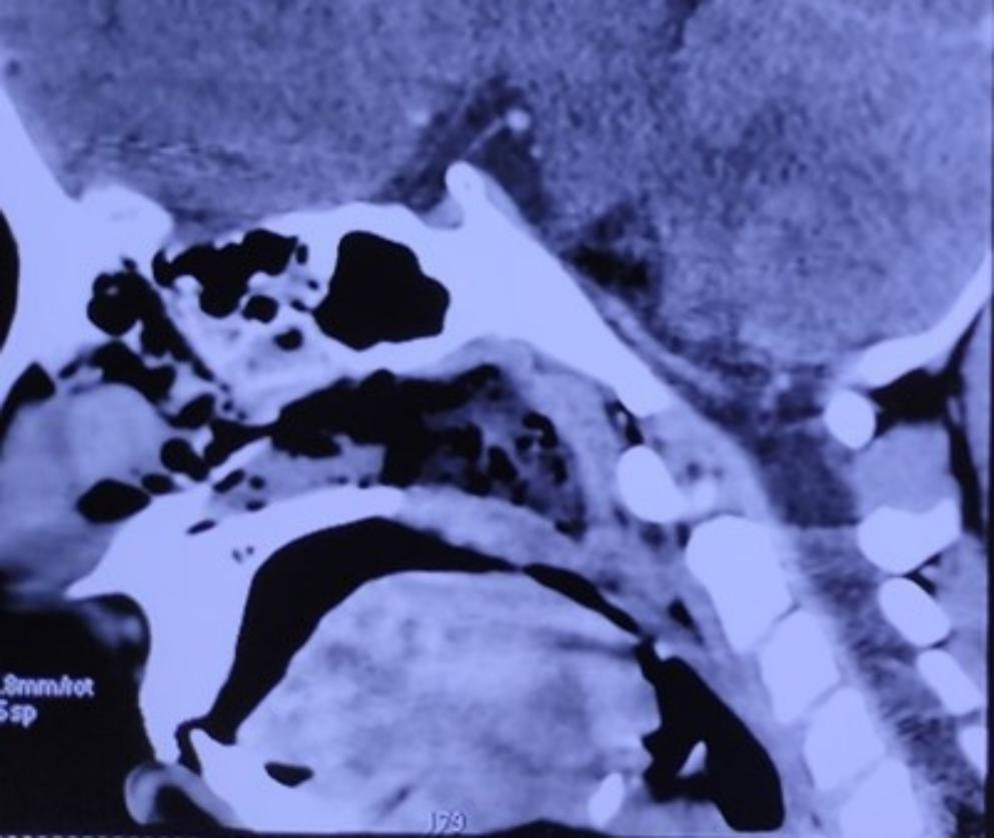
Plain Computed tomographic image of the nasal cavity and nasopharynx sagittal section showing complete obstruction at the nasopharynx

**Fig. 2:**
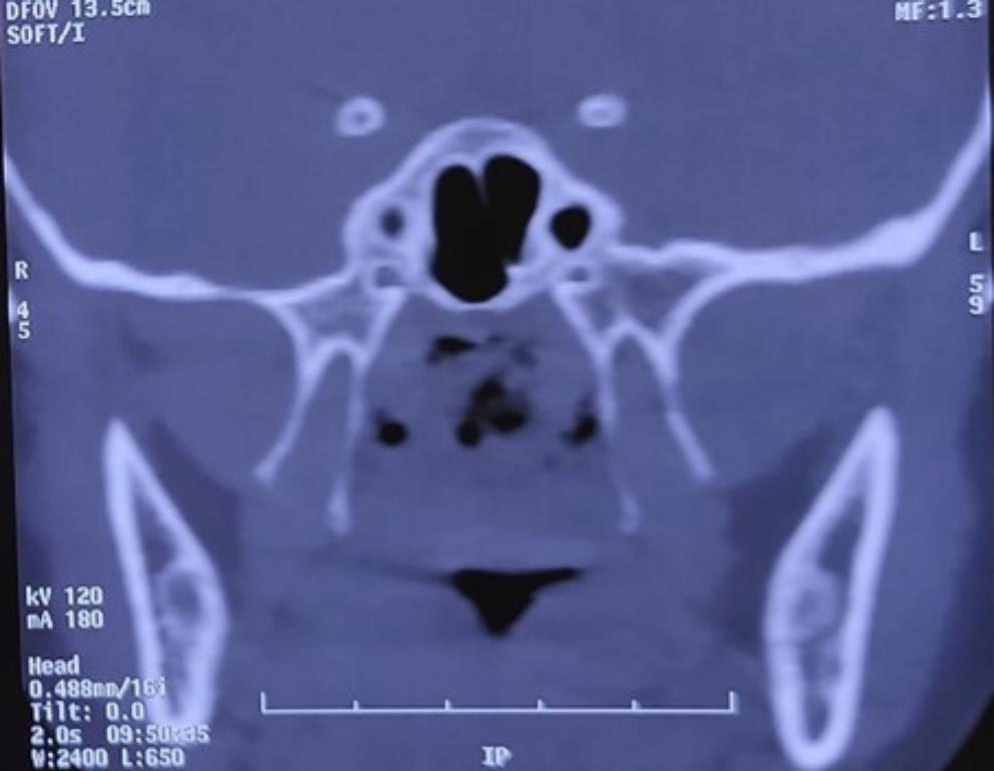
Plain Computed tomographic image of the nasopharynx coronal section showing complete obstruction at the nasopharynx

A 9 year girl child presented with complains of nasal obstruction since 3 months, associated with snoring, mouth breathing and breathlessness on exertion. H/o adenotonsillectomy 5 years ago. H/o coblator assisted palatoplasty and stricture release 4 months ago. On examination adenoid facies was noted, postpalatoplasty and tonsillectomy status with stricture between the tonsillar pillars. Uvula and soft palate adherent to posterior pharyngeal wall, only 2 mm gap in the region of velopharynx (Grade III-Krespi and Kacker). NECT was done which showed homogenously enhancing soft tissue density in the posterosuperior aspect of nasopharynx with maximum thickness of 6.7 mm (residual adenoid tissue), nonenhancing soft tissue attenuation with fluid density and air foci in nasopharynx 35 × 29 × 17 mm causing complete nasopharyngeal airway obstruction (Figs. 1 and 2).

**Fig. 3:**
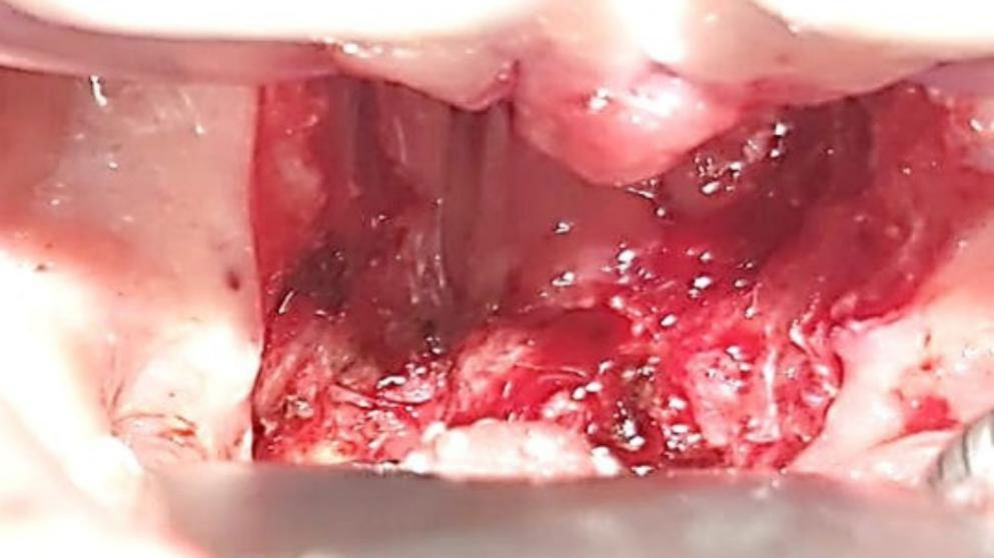
Intraoperative image showing the incisions given at the lateral pharyngeal wall to release the strictures on both sides

**Fig. 4:**
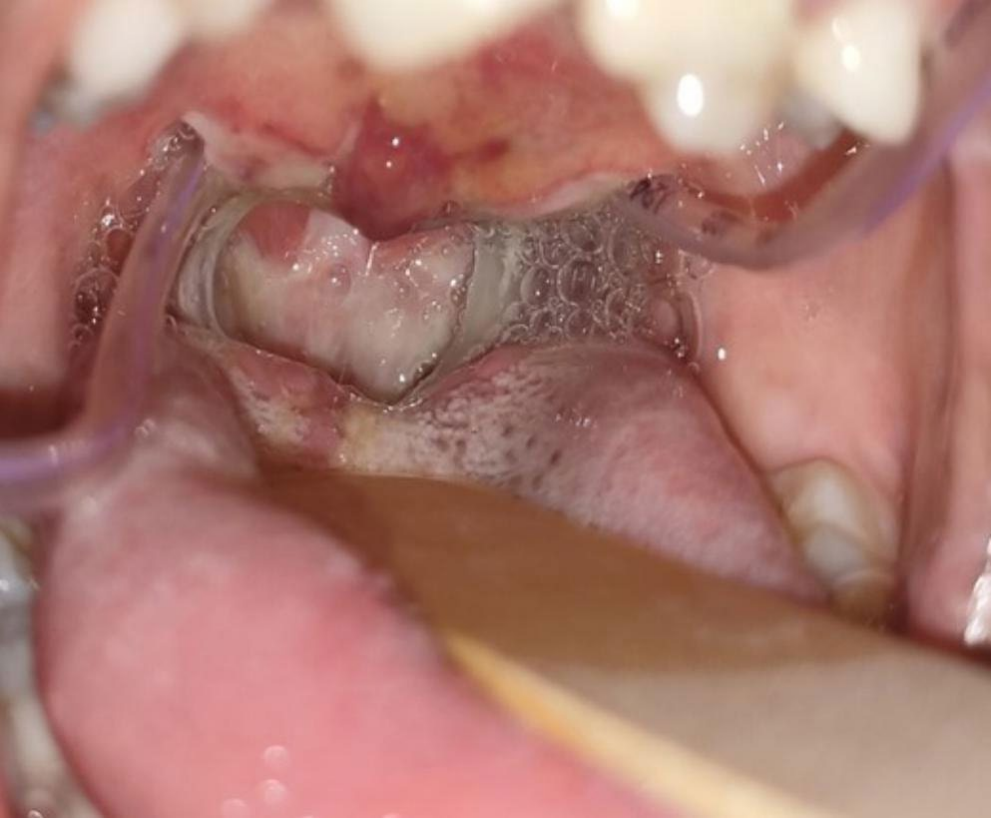
Postoperative day 2 image showing the patent oropharynx along with infant feeding tube in situ

**Fig. 5:**
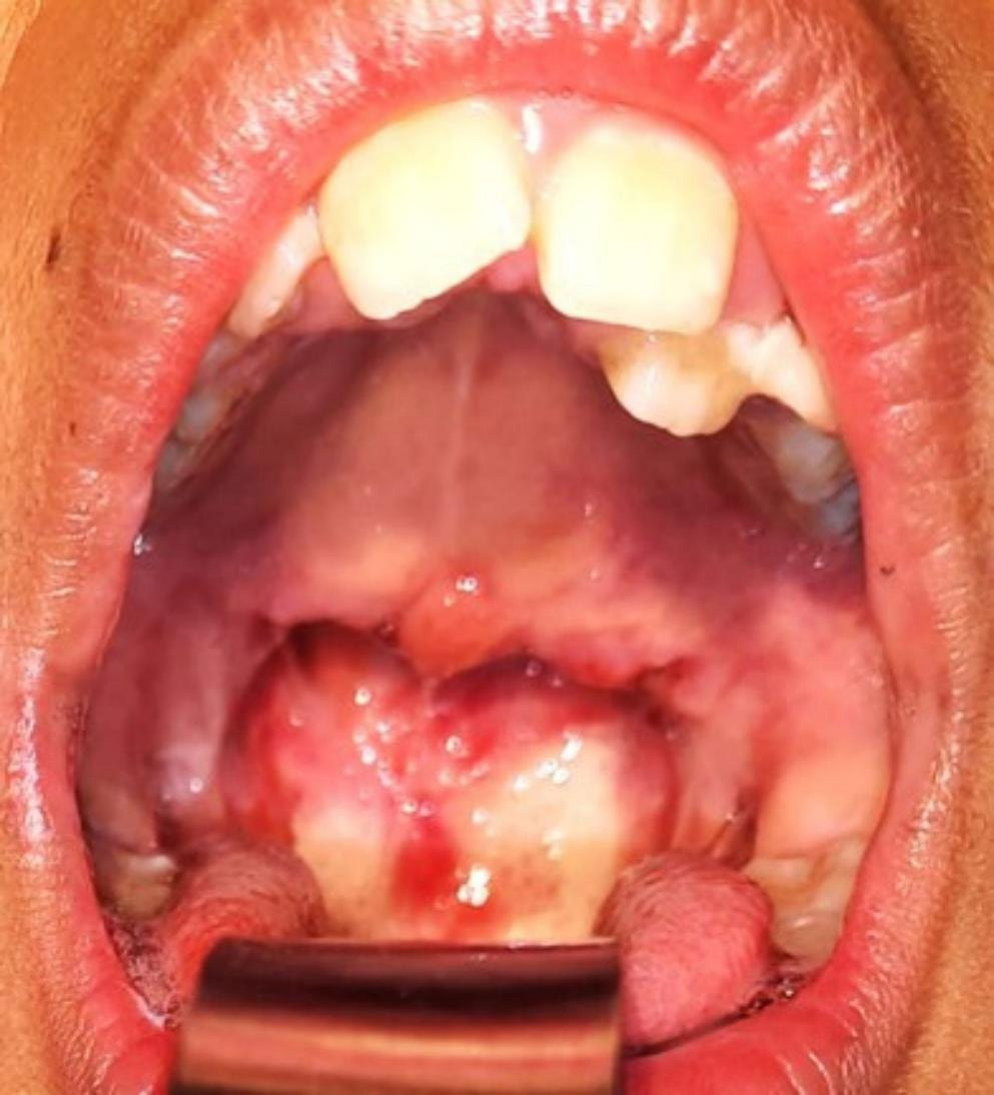
Postoperative day 10 image showing the patent healing oropharynx

Oronasopharyngeal stricture release under general anesthesia was performed scalpel and coablator wand. Hemostatic Feracrylum gel 3% was used over the raw surfaces and infant feeding tube was inserted through the nasal cavity and introduced through the oropharynx into exterior and was tied externally which was removed on postoperative day 7. Child is on regular follow up since 5 months and asymptomatic (Figs. 3, 4 and 5).

## Discussion

Oropharyngeal stricture is reported as a rare sequela of adenotonsillectomy. Dissecting of the lower pole of the tonsil deeply, excision of nearby lingual tonsillar surfaces lead to stricture formation. Excessive use of cautery and immediate postoperative hemorrhage is usually associated with oronasopharyngeal stricture [4].

A classification system of nasopharyngeal stenosis was suggested by Krespi-Kacker. Type I is the mildest form in which lateral regions of the soft palate gets stuck to the posterior pharyngeal wall. In type II stenosis, there is circumferential scarring with a central patency of around 10 to 20 mm. Type III stenosis is the severe type, in which an opening of < 10 mm will be noted [5].

During the surgical release of scars and bands, the mucosal surfaces that are injured and exposed raw, may lead to further scar formation and subsequent formation of restenosis. Many methods have been described in the previous articles to resurface the raw surfaces and thereby prevent healing of the raw areas by secondary intention. Purastat, a novel haemostatic material was used intraoperatively in nasopharyngeal stenosis patient successfully [6]. We used simple hemostatic Feracrylum gel 3% and infant feeding tube was used to prevent apposition of raw surfaces. No restenosis was observed in our case till date.

Surgical procedure is the usual source of Oronasopharyngeal stricture, cases with smaller upper aerodigestive tract, specifically pediatric cases should be carefully assessed for appropriate surgical technique prior to any surgical procedure. Utmost care must be taken to prevent collateral damage to mucosal surfaces which can result in such devastating complications.

## Conclusion

Utmost care must be taken during the surgical procedure of oropharynx to prevent collateral damage to mucosal surfaces which can result in such devastating complications.

## Electronic Supplementary Material

Below is the link to the electronic supplementary material.

Supplementary Material 1
