# Supplementary figures and images for: Pediatric Oronasopharyngeal Stricture– A Rare Surgical Complication of Adeno-Tonsillectomy Abstract
Source: Indian J Otolaryngol Head Neck Surg. 2023 Mar 20;75(3):2352–4. doi: 10.1007/s12070-023-03694-5 (PMC10447637; doi:10.1007/s12070-023-03694-5)

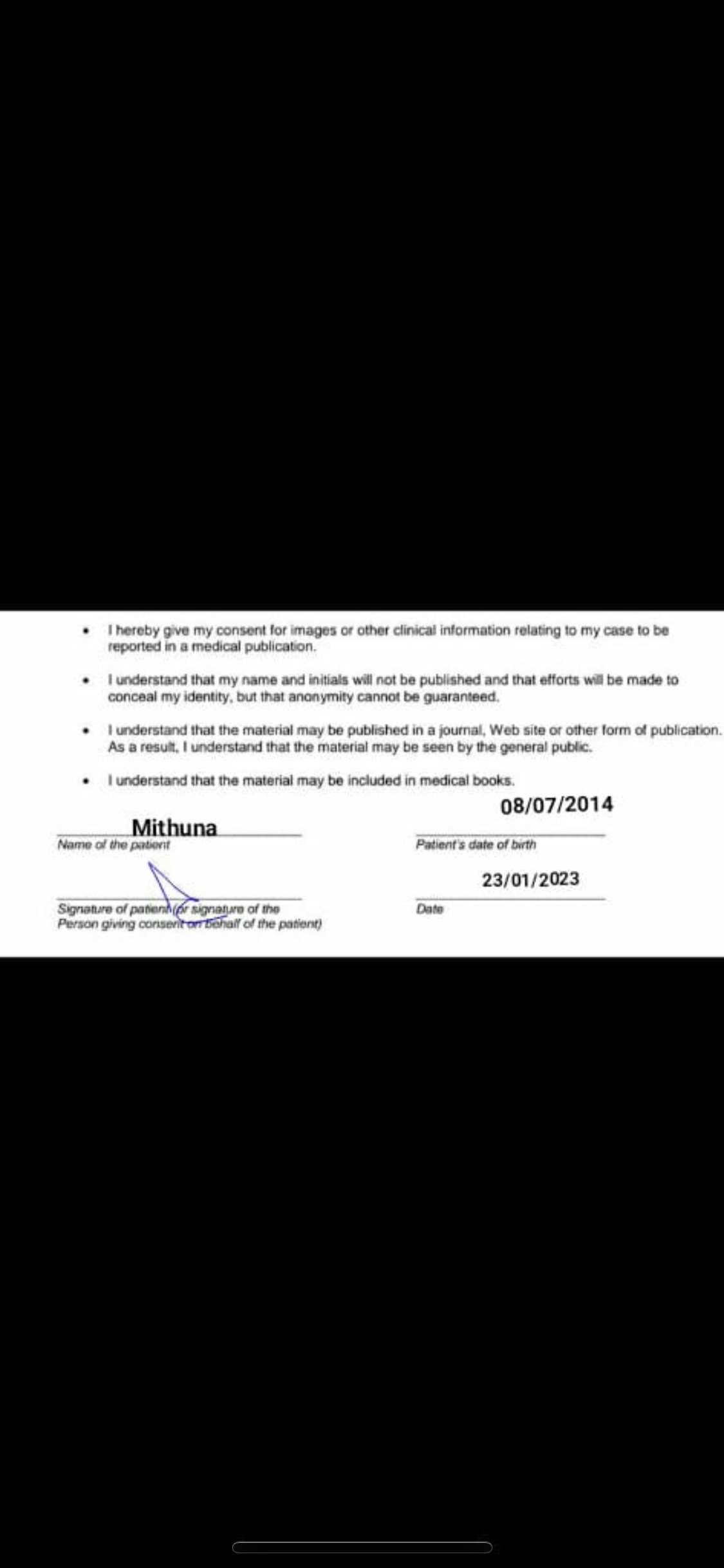

Supplement: Supplementary file 1 — Supplementary Material 1 [file 12070_2023_3694_MOESM1_ESM.jpg]
